# The timing of storm awareness in the Caribbean: the utility of climate information for improved disaster preparedness

**DOI:** 10.1111/disa.12540

**Published:** 2022-06-07

**Authors:** Denyse S. Dookie, Jacqueline Spence‐Hemmings

**Affiliations:** ^1^ Research Officer, Grantham Research Institute on Climate Change and the Environment London School of Economics and Political Science United Kingdom; ^2^ Climate Branch Head, Meteorological Service Division Ministry of Economic Growth and Job Creation Jamaica

**Keywords:** Caribbean, climate information, disaster communication, disaster preparedness, storm awareness

## Abstract

Noting the frequency of tropical storm hazards and related disasters within the Caribbean, this paper initially highlights the relatively short average period of ‘storm awareness timing’ in the region, less than 24 hours, with variations in time and space. Next, it evaluates the results of a survey on communicating disaster risk by a range of participants at the 2016 Wet/Hurricane Season Caribbean Community Climate Outlook Forum in Dominica. Respondents commented that there may be a ‘weekend effect’ possibly hindering quick action and suggested that improved institutional support was needed to use climate information better. Analysis of these two datasets in tandem offers a unique understanding of whether the timing of events may contribute to limitations on responses by local authorities. Lastly, the paper ends with insights into how this research can assist regional authorities in enhancing and utilising climate information for disaster risk reduction, as well as by indicating where critical issues remain.

## Introduction

Historically, tropical storms have wreaked havoc on Caribbean islands, affecting millions of people, causing billions of dollars of economic damage, and disrupting the development process in inherently vulnerable states. In the past five years (2016–20) alone, a total of nine tropical cyclones have struck 21 Caribbean countries and territories, impacting on 13.5 million persons in total and causing an estimated USD 89.6 billion in economic damage.^1^ Some of these storms, including Hurricanes Irma and Maria in 2017 and Hurricane Dorian in 2019, were particularly catastrophic for many of the Caribbean's vulnerable Small Island Developing States (SIDS), challenging post‐disaster recovery plans and development priorities.

While the Caribbean is no stranger to tropical cyclones, disaster management efforts are tested in times of intense storms. Importantly, such events are brutal reminders that disaster preparedness and resilience remain essential elements of the disaster risk reduction process: ‘[i]nvesting in resilience *before* a disaster hits is more effective than relying heavily on post‐disaster recovery’ (Upperman et al., 2019). However, while ‘building back better’—a thoughtful and thorough approach to strengthening resilience (UNDRR, 2022a)—has been touted as a critical forward‐thinking strategy, ‘there has been little serious reflection on the types of action needed for long‐term resilience’ (Wilkinson, Twigg, and Few, 2018, p. 2).

It is certain that the Caribbean Disaster Emergency Management Agency (CDEMA) has undergone institutional reforms that are poised to improve management and reduce disaster risk (Dookie and Osgood, 2021), enhanced by the creation of a Regional Training Centre aimed at building regional capabilities for comprehensive disaster management (CDM).^2^ Furthermore, regional efforts such as the Caribbean Climate Outlook Forum (CariCOF) aim to enhance awareness and development of regional‐relevant climate services focused on adaptation to climate variability and change and disaster risk reduction, encouraging dialogue among stakeholders from the scientific community, disaster management agencies, and key climate‐sensitive sectors, including agriculture, health, and water resources management.^3^ Yet, there is a tendency for these efforts to be generally event‐driven or reactive to the impacts of disasters (Collymore, 2011), with limited proactive preparedness. In a review of preparedness and disaster management plans within the region, Wilkinson et al. (2021b, p. 17) noted that ‘many are not up to date and testing of plans is severely limited … [reflecting] the capacity constraints and variations between Caribbean countries’. Concerning climate‐based natural hazards, ‘actions taken when extreme weather is forecast are ad hoc and incomplete’ (Wilkinson et al., 2021a, p. 1).

Within the comprehensive context of ‘building back better,’ work plans and recommendations that encourage a focus on preparedness and resilience showcase a range of related insights, such as preparedness planning, early action protocols, impact‐based forecasting, forecast‐based early action, and anticipatory action (Wilkinson et al., 2021b, 2021a). Central to these is the need for improved awareness of local contexts, including data on hazard risks, forecasts, and other climate information, to support actions in advance of disasters. While there have been significant research advances in the meteorological and climatological spheres relating to storm development and forecasting, and notable progress in terms of policy scrutiny of the interventions necessary for improved disaster management, understanding of storm risk communication, as well as the time frames for preparedness and early action, has remained relatively limited.

As a starting point, this paper utilises available climate information in a unique way to comprehend how countries may be affected differently by storm disasters. It does this by first synthesising historical observational data based on storm forecasts (watches and warnings) and storm tracks to create a dataset on ‘storm awareness timing’ in the Caribbean. We define this as the calculated difference in time between the first issuance of a forecast of a storm for a particular island and the time when that storm ultimately made landfall or made a close approach. Next, the paper ventures into a discussion of storm risk communication and whether the timing of events contributes to possible limitations to the responses of local authorities within the region. This is done through an evaluation of a survey of participants at the Wet/Hurricane Season CariCOF in Dominica in May 2016.

Our analysis of the quantitative storm data reveals that while there is a range of storm awareness timings across the islands, eight of the 14 countries included in our sample are aware of impending threats within 24 hours of impact/approach on average, while one country, Grenada, receives information within 12 hours of impact/approach on average. We consider this result in the context of the findings of our qualitative survey data, which highlight that information awareness and dissemination is generally slower on a weekend due to capacity limitations. This juxtaposition emphasises the often‐imminent threat of storms within the region and offers some justification and motivation for improving regional and national preparedness and resilience efforts. In particular, there is an acute need to understand better the communication of storm risk within the region, and specifically how technology and data, including climate information, can assist the preparedness process. The paper concludes by offering some insights into what these results may imply and how they may assist regional and national preparedness and resilience efforts, especially with regard to using available climate information more effectively.

## Storm awareness times in the context of disaster preparedness in the Caribbean

Since the Latin America and Caribbean region is the second most disaster‐prone region in the world (UN OCHA, 2020), a growing collection of studies have highlighted the nature of and the challenges posed by natural hazard‐based disasters. Increasingly, this work has centred on the insular Caribbean, given the compounding issue of vulnerability due to small physical, demographic, and economic size (Bello et al., 2017). Research in this regard has focused on the wide‐ranging impacts of storms, drought, and seismic disasters on a country's economic situation, including debt, production, growth, tax revenue, fiscal response, and damage and losses (Pielke, Jr. et al., 2003; Rasmussen, 2004; Heger, Julca, and Paddison, 2008; Hsiang, 2010; Strobl, 2012; Robinson and Bangwayo‐Skeete, 2016; Bello, 2017; Mohan and Strobl, 2021), as well as on the human ramifications, migration, and differential responsibility (López‐Marrero and Wisner, 2012; Spencer and Urquhart, 2018). Within this scholarship, many studies specifically spotlight the character and consequences of storms within the region, probably as a result of the severity of the effects of hurricanes on economic and social development in Caribbean countries. In addition, a plethora of scientific research on tropical cyclones in the Atlantic Ocean exists, concentrating on activity and climatology (Walker et al., 1991; Landsea, 1993; Burn and Palmer, 2015), prediction and forecasting (Landsea and Cangialosi, 2018; Camargo et al., 2021), and changing nature due to global warming (Knutson et al., 2010; Campbell et al., 2011; Mendelsohn et al., 2012; Emanuel, 2017).

In light of the wider set of natural hazard‐based disasters, it is not surprising that efforts to improve understanding and design of disaster risk management strategies in the Caribbean have been evolving over time. Collymore (2011) has offered broad insights into the reform and development of the institutional capacity to inform disaster management within the region, noting that fundamental changes were needed especially in terms of internal capacity development rather than a reliance on external interventions, as well as to be less mono‐focused and response‐oriented. Preparedness, then, or the development of ‘knowledge and capacities developed by governments, response and recovery organizations, communities and individuals to effectively anticipate, respond to and recover from the impacts of likely, imminent or current disasters’ (UNDRR, 2022b), is considered to be an essential element of disaster risk reduction that still requires further effort within the region (Charvériat, 2000; Rasmussen, 2004; Pelling, 2010; Kirton, 2013; Chmutina and Bosher, 2015; Murray and Watson, 2019; Wilkinson et al., 2021a).

**Figure 1:**
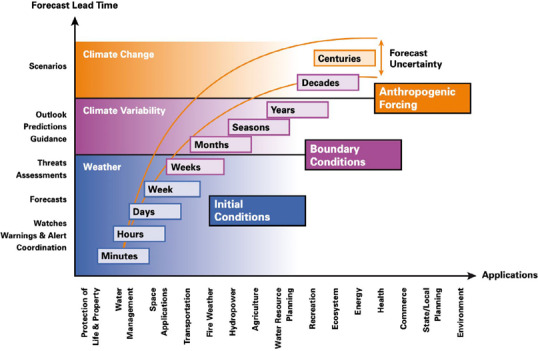
Climate information timelines, products, and applications **Note**: The schematic summarises possible climate information products and their applications, highlighting gaps in developing a balanced and seamless approach to weather, climate, and water products and services. **Source**: WMO (2011).

In this context, efforts to improve technology and data, including climate information, can be a necessary source of relevant insights contributing to disaster preparedness and an organised transition from response to recovery (Vermeiren and Watson, Jr., 1994; Dookie, Enenkel, and Spence, 2019; Dookie and Osgood, 2021). Climate information can be defined broadly as data on historical, current, and future weather and climatic conditions in a specific locale along different timescales for decision‐making, combining scientific and traditional knowledge (Dinku et al., 2011; Singh et al., 2018). As shown in Figure 1, there is a broad range of possible climate information along varying timescales, from shorter‐term advisories, including watches, warnings, and other alerts, to medium‐term forecasts and outlooks, to longer‐term scenarios, as highlighted along the vertical axis. These data can be used in numerous ways in multiple sectors, as shown on the horizontal axis. While Figure 1 illustrates the wide array of options, the verification and effective utility of such products in the Caribbean region is an ongoing topic of research (Dookie, Enenkel, and Spence, 2019). Nevertheless, the value of such weather and climate information is regarded as essential for disaster preparedness and risk management and adaptation to climate variability and change. In an analysis of the integration of disaster risk management policies into development policies in five Caribbean countries (The Bahamas, Belize, Dominican Republic, Haiti, and Jamaica), Bello et al. (2017) specifically underscored the importance of updated and readily available data for decision‐making. While the need for such information is recognised (Mahon et al., 2019), the paper offered minimal guidance on access to this data and the collation, dissemination, and effective use of it within the selected countries, even though it ‘would allow line ministries to identify exposed assets and vulnerable populations, and take actions to mitigate or reduce the risk of disaster’ (Bello et al., 2017, p. 66).

This paper looks at how we can utilise climate information—such as storm advisories, that is, tropical storm and hurricane watches and warnings—better to assist preparedness in the context of disaster risk management. A ‘watch’ announcement is issued when tropical storm or hurricane conditions are *possible* within the specified coastal area within 48 hours, whereas a ‘warning’ announcement is issued when tropical storm or hurricane conditions are *expected* within the specified coastal area within 36 hours (since the hurricane season of 2010). The United States’ National Hurricane Center (NHC), as part of the National Oceanic and Atmospheric Administration (NOAA), continuously monitors and gauges the verification and performance of storm advisories and forecasts. In fact, improvements in NHC forecast track errors have led to advisories now being issued 12 hours earlier than was the case in the past.^4^

Furthermore, forecasts for Atlantic basin cyclones have been improving in terms of accuracy of intensity (Wilkinson et al., 2021a). While meteorological research has expanded over time, the associated focus on the utility of such forecasts, and in particular the timeliness of information, for preparedness and resilience actions is not yet as evident within the literature. One relevant paper in this regard looks at the trade‐off between forecast accuracy and the timeliness of information via shorter lead times for decision‐making in the southeast US (Regnier, 2020). However, for a vulnerable region such as the Caribbean with limitations on financial, technical, and human resources that can affect preparedness measures, two critical questions are not readily answered in the literature:

- How long do we have before a storm hits or makes an impact?
- Does the time vary by country?

So, we seek to advance the discussion on the relevance of climate information for enhanced disaster preparedness by specifically reviewing observational data for the Caribbean. We review a compiled dataset of ‘storm awareness times’ for the region (Dookie and Osgood, 2020). Storm awareness time (the original research refers to this as ‘storm lead time’) is defined here as the difference in time, in hours, between the first forecast (watch or warning) of a storm for a particular country, and the time of impact of landfall or approach of this storm for the same country. While it may be that the wider public may focus only on later forecasts leading up to impact, this dataset concentrates on occurrence of the first advisory, whether this is a tropical storm or hurricane watch or warning, since we assume this to be the starting point of awareness of the existence of an impending threat, and subsequent monitoring and activation of preparedness measures. To offer a broad context of storm impact, the dataset considers both storm ‘landfall’ (that is, when the eye of a storm crosses a country's coastline) and close approach, broadly referring here to whether the eye of the storm came within 100 nautical miles of the coastline, which includes storms that entailed a ‘direct hit’ (that is, when the core of high winds (or eyewall) comes over the land, but the centre of the storm stays over the water). This is because some storms may not officially make landfall or have their eyewall over land but may still be quite large and consequential. As such, the difference in time between the first advisory of the storm and its impact upon landfall or approach could be interpreted as the known time for preparedness actions to be taken or implemented.

Data were primarily compiled for Caribbean countries affected by either tropical or convective storms within the region in the selected period, and for which there was readily available storm forecast advisories as well as supplementary economic and disaster impact data. Given these criteria, the dataset centres on 14 Caribbean countries: Antigua and Barbuda; The Bahamas; Barbados; Cuba; Dominica; Dominican Republic; Grenada; Haiti; Jamaica; Puerto Rico; Saint Kitts and Nevis; Saint Lucia; Saint Vincent and the Grenadines; and Trinidad and Tobago. Storm awareness time data were calculated using storm forecast advisory data extracted via web scraping and manual review of tropical storm watches and warnings as issued by the NHC on behalf of national governments,^5^ as well as storm landfall/approach data collated from the NOAA website on historical hurricane tracks, based on the NOAA NHC's HURDAT2^6^ and the NOAA National Centers for Environmental Information's IBTrACS^7^ datasets.^8^

For a broader understanding of how storm hazards have affected the region, the original dataset also reviewed disaster data in the Centre for Research on the Epidemiology of Disasters’ Emergency Events Database (EM‐DAT),^9^ for which at least one of the following criteria must be fulfilled to be considered as a disaster event: 10 or more deaths; 100 or more people affected, injured, or made homeless; or a declaration by the country of a state of emergency and/or an appeal for international assistance. Using markers such as event name, date, and country, corroboration was made between the storm awareness time dataset (based on storm forecast and landfall/approach) and the disaster event dataset to arrive at a comprehensive dataset of 486 event occurrences in the Caribbean in the selected period.

**Figure 2:**
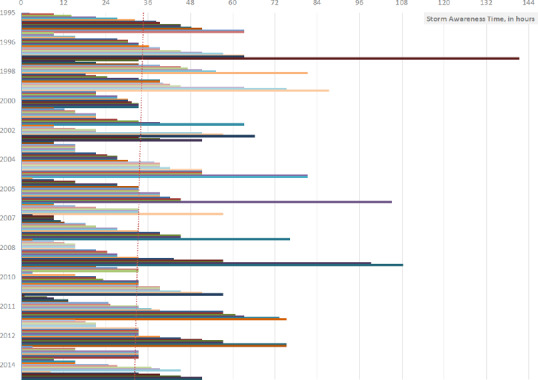
Summary of storm awareness times for 14 Caribbean countries, 1995–2015 **Notes**: each bar represents the storm awareness times, in hours (horizontal axis), for 251 storm events that had a forecast and made an approach/landfall across 14 Caribbean countries in the years 1995–2015 (vertical axis)—for example, the longest storm awareness time in this dataset is 141 hours for an event in 1996. Note that there was at least one storm event in each year, but this is not reflected due to some years having many events. This calculation is made using data from the NHC website and the NOAA website on Historical Hurricane Tracks using the NHC's HURDAT2 and the NOAA's National Centers for Environmental Information IBTrACS datasets. The storm awareness time is defined here as the time difference, in hours, between the first storm advisory issued (forecast watch or warning) for a particular storm for a particular country and its approach/landfall. Figure 2 only includes occurrences of a storm having both a forecast and an approach/landfall, and the average storm awareness time for these 251 events is 33.4 hours. **Source**: authors.

The first observation regarding these calculations is that there is a wide range of storm awareness times over the period. Figure 2 illustrates the varying nature of storm awareness times using compiled data for 251 storm events that had both an issued storm advisory and made an approach/landfall (52 per cent of 486 total observations). Each bar in Figure 2 represents an individual storm awareness time, in hours (horizontal axis), for 251 storm events that had a forecast and made an approach/landfall across 14 Caribbean countries between the years 1995 and 2015 (vertical axis). For example, the longest bar corresponds to the longest storm awareness time in this dataset: 141 hours for an event in 1996. For the 14 selected countries in the period 1995–2015, the average storm awareness time was 33.4 hours, within a broad range of 0.5–141 hours. This average seems to be slightly decreasing over time, as indicated by the dotted trend line.

Second, the calculations reflect that there were a number of ‘false alarms’ as well as ‘zero‐lead’ events. A ‘false alarm’ was indicated whenever an advisory was issued for a storm that did not ultimately make an approach or landfall. A ‘zero‐lead’ was indicated whenever there was a storm that made impact, as evident from the storm trajectory dataset based on Historical Hurricane Tracks or EM‐DAT, but no corresponding storm advisory was issued. For instance, Tropical Storm Erika was destructive on the island of Dominica in August 2015 but there was no issued forecast; best‐track models did not forecast the storm making landfall and no official advisory was issued to Dominica as it was to other Leeward Islands, but the storm came close to the small island and the wide rain and wind bands were indeed able to cause devastating damage (there is a disaster event entry for this event within the EM‐DAT dataset). Another example is Hurricane Michelle, which impacted Jamaica in October 2001; while the storm track shows that Michelle never even came physically close to the island and advisories were not issued, satellite rainfall estimates indicate torrential downpours as a result of the passing system and there is a recorded entry within the EM‐DAT dataset. The compiled storm awareness time dataset indicates a zero‐lead event with an entry of zero hours, indicating that there was no apparent awareness of the impending storm threat based on official forecasts.

In total, there were a recorded 102 false alarms (21 per cent of the total 486 observations) and 133 zero‐lead events (27 per cent of the total 486 observations) for the 14 selected Caribbean countries in the period 1995–2015. The number of false alarms and zero‐lead events per country is tallied in Table 1; countries within the Greater Antilles (such as The Bahamas, Cuba, Dominican Republic, and Haiti) seem to have a relatively higher number of zero‐lead events, but this could be a function of the high number of impacting events in those countries. In fact, Grenada has the highest zero‐lead event ratio within the region, based on impacting events, followed by Dominica. In terms of false alarms, Trinidad and Tobago received the most per total observations, but this could be due to the caution applied to the southern‐most Caribbean island, which lies just south of the typical hurricane belt.

**Table 1:** Storm awareness timing, by country, 1995–2015

	**Total recorded observations**	**Number of impacting storms**	**Storm awareness average (hours)**	**Maximum awareness (in hours)**	**Weighted average**	**Number of zero‐leads**	**Number of false alarms**
**Antigua and Barbuda**	36	28	26.6	87	0.95	7	8
**The Bahamas**	60	50	31.2	108	0.62	14	10
**Barbados**	21	17	13.3	33	0.78	4	4
**Cuba**	50	44	27.6	141	0.63	16	6
**Dominica**	34	23	14.8	51	0.64	10	11
**Dominican Republic**	47	40	17.2	57	0.43	16	7
**Grenada**	24	20	9.0	42	0.45	12	4
**Haiti**	46	44	19.1	66	0.43	15	2
**Jamaica**	33	21	27.0	63	1.29	7	12
**Puerto Rico**	38	24	28.4	63	1.18	7	14
**Saint Kitts and Nevis**	32	24	26.3	75	1.10	7	8
**Saint Lucia**	26	19	15.2	39	0.80	7	7
**Saint Vincent and the Grenadines**	26	22	14.4	39	0.65	8	4
**Trinidad and Tobago**	13	8	12.0	33	1.50	3	5
**Regional**	486	384	21.8	141	–	133	102

**Notes**: Table 1 summarises information for 486 recorded observations collated across 14 Caribbean countries between 1995 and 2015, using data from the NHC website, the NOAA's Historical Hurricane Tracks website, and EM‐DAT. Number of impacting storms is the tally of observations for storms that were forecast and ultimately made an approach/landfall. Storm awareness average is the average per country of the storm awareness time defined as the difference in time, in hours, between the first forecast (watch or warning) of a storm for a particular country, and the time of impact of landfall or approach of this storm for the same country. This includes zero‐leads or storm events for which there was impact but no advisory with a resulting ‘0‘‐hour storm awareness time. Maximum awareness is the longest storm awareness time per country. Weighted average is the weighting of the storm awareness average per country by the number of impacting storms. Number of zero‐leads is the tally of storm events for which there was impact but no advisory. Number of false alarms is the tally of storm events with an advisory but no impact.

**Source**: authors.

A third key observation concerns the range of storm awareness times across space. Table 1 also indicates the distribution of impacting events and storm awareness times in each of the 14 selected Caribbean countries. Column 3 of Table 1 sums the number of impacting events and is the difference between total observations and the number of false alarms. Of the total number of 486 observations recorded in the original dataset, a sum of 384 storm events had some type of impact, whether or not there was advance awareness in the form of at least one storm advisory. As shown, and not surprisingly, countries within the Greater Antilles had the highest number of impacting events. We wish to note at this point (discussed further below) the importance of considering the general dynamics of storm development and trajectories, as storms usually develop out of tropical waves brewing in the eastern Atlantic Ocean around Cabo Verde and owing to the Coriolis force move in a northwest direction as they approach the islands of the Caribbean. Column 4 of Table 1 highlights the differences in storm awareness times across the region, considering the sample of impacting events with overall awareness times ranging from 0 to 141 hours (column 5). Across the countries, there was a range of average awareness of between 9 (Grenada) and 31.2 (The Bahamas) hours, for an overall regional average of 21.8 hours. This regional average is contrasted with the aforementioned 33.4 hours for events that had both an advisory and an approach/landfall, that is, not considering zero‐lead events.

**Figure 3:**
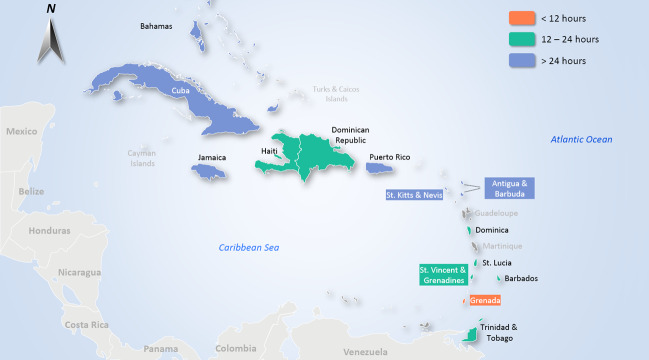
Map of Caribbean islands, based on average storm awareness time **Note**: map based on Table 1, illustrating column 4, the storm awareness average (in hours) by country. **Source**: authors, using the map template at https://www.presentationload.com/map-caribbean-islands-powerpoint-template.html (last accessed on 5 May 2022).

**Figure 4:**
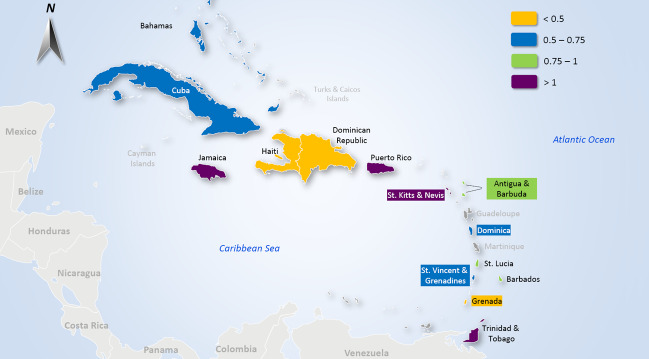
Map of Caribbean islands, based on storm awareness time weighted by the number of impacting storms **Note**: map based on Table 1, illustrating Column 6, the weighted storm awareness average (in hours) by the number of impacting events, by country. **Source**: authors, using the map template at https://www.presentationload.com/map-caribbean-islands-powerpoint-template.html (last accessed on 5 May 2022).

To help visualise this data, Figure 3 (above) indicates the variation in storm awareness times across the selected Caribbean islands. The map shows that there are eight (of the selected 14) countries with less than 24 hours average lead time (that is, most of the Lesser Antilles, Dominican Republic, and Haiti), with Grenada being the only country with less than 12 hours average storm advisory lead time. To add further context to this information, Figure 4 (above) utilises the weightings calculated in Table 1 (see column 6) to present a map with storm awareness times weighted by the number of storms that have affected each island (based on relevant available information as per this dataset). Dominican Republic, Haiti, and Grenada stand out as the countries most affected by short storm awareness times, followed by The Bahamas, Dominica, Cuba, and Saint Vincent and the Grenadines.

We provide some context and discussion of these observations below, following a review of a qualitative dataset of disaster risk communication within the region.

## Communicating disaster risk within the Caribbean

Exacerbating the challenge of short storm awareness times on disaster preparedness, and the general need for encouraged use of climate information, we highlight now a regional concern of communicating disaster risk within the region. At the Wet/Hurricane Season CariCOF in Dominica in May 2016, participants were asked to complete a survey relating to the communication of climate information within the region (see Box 1). Respondents comprised regional and country representatives of meteorological offices/services (Met Offices) and disaster agencies as well as specialists from the water, tourism, and agricultural sectors, offering a wide panorama of the regional context. The survey was discussed within groups based on table seating arrangements before completion per table.

As shown in Box 1, in response to a survey question relating to whether or not there were adequate resources within weather and disaster agencies on a weekend, as compared to on a weekday, there was wide agreement that there was a ‘weekend effect’ that negatively affected capacity. In offering thoughts on the related reason for this, selections included challenges on the public side (communication with/within the local public may be limited on a weekend due to cultural, recreational norms), and on the agency side (including that some agencies are closed or at limited capacity, thereby offering slower response times on a weekend, that certain sectors did not maintain a 24‐hour shift system, and that there was a challenge of mobilising volunteers on a weekend). Interestingly, most participants who mentioned that there was a ‘weekend effect’ were from the English‐speaking Caribbean.

**Box 1. Summary results of the CariCOF Expert Feedback Survey**

The purpose of the survey was to understand the effect of weekends on disaster preparedness and the resources needed to improve preparedness within the region. In summary:

- Eleven responses received (the survey was completed by tables of between five and nine persons).
- Nine sectors represented: agriculture; disaster; health; local government; media; meteorology/climatology; research/university; tourism; and water.
- Four agencies represented: Caribbean Institute for Meteorology and Hydrology; Caribbean Meteorological Organization; Caribbean Tourism Organization; and Environment Canada.
- Nineteen countries represented: Antigua and Barbuda; Aruba; The Bahamas; Barbados; Belize; Cayman Islands; Cuba; Curacao; Dominica; Grenada; Guyana; Jamaica; Martinique; Saint Kitts and Nevis; Saint Lucia; Saint Maarten; Saint Vincent and the Grenadines; Suriname; and Trinidad and Tobago.
- Response to ‘Do you think the weekends/holidays affect the state of disaster preparedness in your country?‘
  - Yes: seven respondents (64 per cent of the total).
  - No: two respondents (18 per cent of the total). Note: one of these responses included the comment that weekends/holidays were of concern.
  - No answer: two (18 per cent of the total)
- Mean response: ‘What is the likely weakest link in disaster/event preparedness?’ (ranked out of ‘5’):
  - 2.88/5: challenges in agency capacity to acknowledge and share initial information about a disaster;
  - 2.88/5: challenges in agency capacity to follow up on information and respond to a disaster;
  - 2.88/5: limited awareness of impending threat by local public (not following news, away from information sources);
  - 2.88/5: limited general preparation for disasters by local public (does not happen often, will not affect them); and
  - 1.00/5: other: timely receipt of appropriate information.
- Mean response: ‘What is the most essential resource needed to assist this weak link?’ (ranked out of ‘10’):
  - 8.14: improved institutional support to utilise this information;
  - 7.60: communication, such as need a better strategy or local coordination;
  - 7.14: networking with other agencies, local and/or regional;
  - 6.86: financial, such as need access to or an increase in funds, either generally or within the responsible agency;
  - 6.75: technical skills of responsible agency;
  - 6.14: human, such as need more capacity within the responsible agency;
  - 5.71: technical assistance, that is, unsure of how to fit this information into existing decision‐making strategies;
  - 5.00: more data, such as climate information, number of people affected, and most vulnerable locations; and
  - 3.43: information on disaster management/best practices/research about disasters within the region.
- Median response: ‘What is the most essential resource needed to assist a weak link?’ (ranked out of ‘10’):
  - 9: communication, such as need a better strategy or local coordination;
  - 8: financial, such as need access to or an increase in funds, either generally or within the responsible agency;
  - 8: improved institutional support to utilise this information;
  - 7: networking with other agencies, local and/or regional;
  - 7: technical skills of responsible agency;
  - 6: human, such as need more capacity within the responsible agency;
  - 5: technical assistance, that is, unsure of how to fit this information into existing decision‐making strategies; and
  - 4: more data, such as climate information, number of people affected, and most vulnerable locations;
  - 3: information on disaster management/best practices/research about disasters within the region.

**Source**: authors.

In terms of considering a likely weak link in communicating disaster risk within the region, there was a generally even distribution of selection between challenges related to the general public and agency capacity. Respondents thought that there was altogether limited general preparation for disasters by the local public (the idea being that there was an underlying rationale among members of the general public that disasters do not happen often or would not affect them) as well as limited awareness of an impending threat among the local public (that is, not following news and keeping away from information sources). Furthermore, respondents noted that there were challenges in agency capacity to acknowledge and share initial information about a disaster, as well as to follow up on information and respond to a disaster.

When asked about the most essential resource needed to address these weak links, most participants said that improved institutional support was needed to utilise available climate information, and that there needed to be improved communication, through better strategising and local coordination.

## Discussion

Given the challenge of storm risk and disaster impacts within the insular Caribbean, and the oft‐cited concern of disaster preparedness, this paper examines two datasets to understand if the timing of climate information could play more of a role within enhanced regional disaster recovery and resilience. The first dataset is a quantitative calculation of storm awareness times for 14 Caribbean countries/territories in the period between 1995 and 2015, and the second is a qualitative survey instrument focused on the timing of disaster risk communication within the region, completed by participants at the 2016 CariCOF.

Analysis of the quantitative dataset highlights that the regional average time of storm awareness is only within 36 hours (33.4 hours on average, for storms with both a forecast and impact, and 21.8 hours if zero‐lead events are included), underscoring the crucial need for ongoing and active preparedness. To add detail, if only the 251 storms that had both an official advisory as well as an approach/landfall are analysed, an average of 33.4 hours seems short, yet it is within the bounds of the time frames of possible or expected storm conditions as defined in storm and hurricane watches and warning announcements. However, as indicated by the trend line in Figure 2, this average storm awareness time seems to be decreasing over time, indicating that over the years, gradually less time, albeit slightly, has been accorded to awareness of storms ahead of impact. Furthermore, if the number of zero‐lead storms are included in this average, and we focus on the 384 observations of impacting storms, the calculations reveal that the regional mean awareness time is slightly less than 22 hours. While an analysis of the trend of awareness times for impacting storms seems to be slightly increasing over time, this is because of a decrease in zero‐lead events from 1995–2015. Although a slight decrease in zero‐lead events is welcome progress, combining this knowledge of relatively short awareness times with the notion of a ‘weekend effect’ within weather and disaster agencies, in that there may be limitations to resource availability and deployment and risk communication on a weekend as compared to the weekday, provides critical motivation for active preparedness efforts within the region.

In addition, the analysis shows that there is a variety of storm awareness times across the selected Caribbean countries, as shown in Table 1 and visualised in Figure 3. The findings reveal that many countries within the Lesser Antilles, or southern Caribbean islands, have relatively shorter storm awareness times, while many of the countries in the Greater Antilles seem to have a longer time to prepare. As mentioned, this finding should be considered in relation to how and where storms usually develop, as certain conditions, including high sea surface temperatures, low vertical wind shear, high relative humidity, the absence of land mass, and the spin of the earth and resulting Coriolis force, affect storm trajectories and intensification. Since the majority of storms are mostly disorganised waves developing off the coast of West Africa, it often takes time to understand fully the potential of each rotating system in the context of the presence of necessary storm conditions, which in turn affect reconnaissance and confirmation of storm advisories. As such, it may be difficult generally to ascertain and predict forecasts for systems as they develop, probably resulting in less time on average for southern countries as compared to those in the north. At the same time, the finding that Haiti and the Dominican Republic both seem to have comparatively lower awareness times among the Greater Antilles countries is a point of note and may be worth investigating further. It is important to underscore that this dataset does not attempt to comprehend the mechanisms pertaining to the onset of each storm, but rather focuses solely on historical observations of when the first advisory was issued in the context of calculating how much time a country has had, on average, ahead of a storm impact.

Another factor that should be considered in terms of these country differences is the issuing authority of the advisories, which may affect timing. While the NHC continuously monitors the North Atlantic for systems of interest, and can issue coastal tropical cyclone watches and warnings for the US and its Caribbean territories, it can only offer watch and warning recommendations to meteorological services within Region IV of the World Meteorological Organization (WMO), that is, North America, Central America, and the Caribbean.^10^ Puerto Rico as a US territory likely receives advisories automatically, but the usual process for the rest of the Caribbean is that these recommendations are offered to national governments, which may engage in further consultations and conduct assessments before authorising public advisory guidance. During consultations with Caribbean meteorological agencies about the storm advisory process within the region, it was revealed that there is a process of information transfer between the NHC and local government authorities and meteorological agencies before such forecasts are made publicly available. This is because there could be a need to delay advice and subsequent closures, which could affect when the first advisory is issued and thus the calculated storm awareness time. While each country has a resident National Meteorological and Hydrological Service, some of these agencies may be a part of the local airport authority that has particular responsibility for aviation meteorology (Mahon et al., 2019) and rely on broader weather information and advice from other countries. For example, as far as is understood, Antigua and Barbuda Meteorological Services offers advice to Saint Kitts and Nevis, while the Barbados Meteorological Service offers advice to assist meteorological services at the airports in Dominica and Saint Vincent and the Grenadines. As each nation holds sovereignty and is responsible for its own jurisdiction, there could in theory be delays in relaying information between agencies before an official advisory is issued. Efforts were made to identify some of this information within the original dataset, but it proved difficult to quantify the time spent between the initial NHC guidance and the issued advisory.

This paper does not focus on the particular mechanisms of forecast issuance in each country, but the differences between countries highlight that research on local circumstances is necessary to understand how preparedness efforts could be improved at the national level. It may not be efficient to advocate for an improved approach to preparedness and resilience without a deeper understanding of the national and even subnational challenges of climate information availability, dissemination, and use, as well as risk communication. With regard to the latter, it is often the case that disaster agencies receive advisory details at the same time as the general public, limiting the organisation and timeliness of sharing relevant preparedness information in what is an already critical awareness time. Referring again to the ‘weekend effect’ of reported inconsistent response capacity due to limitations on personnel and awareness on weekends and holidays,^11^ these short storm awareness times exacerbate the challenges of communicating risk across agencies and to the public, and how preparedness measures could affect risk reduction.

The zero‐lead ratios and high false alarm rates across the region and within certain countries are another motivation for more discussions on the need for better storm research and monitoring. Based on the data summarised in Table 1, Grenada and Dominica seem to have the highest zero‐lead awareness ratios when considering the number of impacting storms. This means that compared to the other countries within the sample, these countries often experienced the impacts of storms for which there was no official advisory. While the trend of zero‐lead events seems to be decreasing on average, probably because of improved technological resources and human capacity within the NHC to monitor tropical disturbances and offer informed guidance, this unawareness of climate information for certain countries should be explored further to ascertain information gaps. As for false alarms, while the data show that Trinidad and Tobago had the highest ratio when considering all observations, which could be due to its location, it is important to mention and question the high rates for Puerto Rico, Jamaica, and Dominica. The nature of storm development likely plays a predominant role in their forecasting and the differences in some of these rates, but perhaps there may be some role for the delicate balance of incorporating certainty into forecasting. For instance, if advisories are issued for low‐confidence systems that result in a false alarm, successive false alarms and high false alarm rates can have a ‘cry wolf’ effect that could discourage preparedness efforts and increase complacency. However, if there is excess caution and a need for complete certainty, this can result in delayed advisories, or zero‐lead events at worst. With respect to certainty, it is also noteworthy that while this paper focuses on the first advisory as a defined starting point of awareness, it could be that some—the general public, but perhaps even local authorities—concentrate on the later forecasts just before a storm impacts. Certainly, this brief discussion does not delve sufficiently into this issue and there is a need for more and deeper data to investigate these complex facets. Yet, the findings of this paper may offer a platform for regional and local discussions on how storm awareness can be improved to increase preparedness and resilience.

It is noteworthy, too, that forecast advisory watches and warnings are generally intended to be offered 36–48 hours in advance. However, such overall short time frames may pose a challenge to the effectiveness of preparedness measures available within small and vulnerable countries such as those in the Caribbean. As highlighted within the qualitative survey at the 2016 CariCOF, limited human and technical resources, particularly decreases in capacity on weekends, affect the awareness and communication of risk within the government and other disaster‐related agencies and authorities and among the local public. Such a delay in communication further affects the timely sourcing and mobilisation of necessary resources and equipment, which are already limited in such ‘developing’ countries (not including practical policy and political issues that can impede the efficient distribution of resources). These considerations are increasingly important in the context of forecast‐based financing and wider anticipatory action. Increasingly, international agencies are advocating for improved approaches to protecting lives and livelihoods ahead of hazards. Strategies now encourage the utilisation of climate information such as forecasts and early warnings as triggers for action, including the release of funds and mobilisation of required resources. However, research such as this underscores the complexity of using climate information, as well as the human resource challenges of awareness and capacity in relation to being able to utilise disbursed funds or resources. Although this paper does not concentrate on the possible mechanisms behind the awareness times or logistical capacity issues within local agencies on weekends, it does provide observational evidence of ongoing challenges within the region that should be addressed to improve preparedness and resilience.

We reiterate here that the dataset and this study are focused on the numerical value of storm awareness times and exclude the effect of ongoing development and research into the tracking, intensification, and forecasting of tropical storms, as performed by the NHC. For instance, we note that systems in the deep tropics move faster due to the strength of the subtropical ridge and deep easterly steering winds, which might result in greater forecasting errors in the advisories recommended by the NHC, and thus in the resulting calculation of awareness times. However, this is beyond the control of the NHC and its continued research into said historical climate information will help to enhance the accuracy of forecast advisories over time. Furthermore, there has been a relatively recent change in the way in which the NHC offers recommendations on the issuance of advisories. Noting the need to decrease the lead time for advice to the public about potential threats, in 2017 (after the scope of the dataset), the NHC started to issue advisories for ‘potential tropical cyclones’, or systems that have yet to develop but may bring tropical storm‐ or hurricane‐force winds to land areas within 48 hours (Belles, 2017). While such a change is largely positive, there are few insights that assess the accuracy of such updated forecasts and understand the scope and impact of such a change in the Caribbean.

As further caveats, we note that this dataset does not specify the type of first advisory for better recognition of the difference in awareness time, such as between a tropical storm watch (tropical storm conditions possible for the specified area within 48 hours) and a hurricane warning (hurricane conditions expected within 36 hours for the specified coastal area). Yet, the original dataset does contain some of this data and more investigation might be of interest in subsequent reviews. And since the original dataset only looks at the time of the first advisory to understand the documented point of initial awareness, it does not confirm any actions taken by countries at the onset of the first advisory, nor does it incorporate the evolution of the issued advisories (such as whether there was a rapid intensification, subsequent degeneration, or surprise trajectory of the storm over time). Knowledge of such broader details could offer rich insights into the patterns of storm awareness times over the period and across the region and assist local authorities in understanding better how to respond to and minimise apparent challenges. We are of the view, though, that since this calculation of storm awareness times has not been done before, it offers an initial look at and starts a dialogue on how storms have historically affected the region over time, which could then influence how storm and other climate information may be better utilised for preparedness.

## The utility of climate information for better disaster preparedness

There is sometimes the fallacy within the Caribbean that that there is sufficient time to mobilise disaster procedures or idiomatic sentiments that the storm will not affect the country at all: ‘God is a Vincy, Trini, Bajan …‘. Since there may be more frequent, intense, and rapidly intensifying storms in a warming climate (Pulwarty, Nurse, and Trotz, 2010; Emanuel, 2017), being alert and prepared for storms should be a constant priority for the majority of Caribbean islands, especially during the hurricane season. To showcase the need for timeliness in the context of preparedness, disaster risk reduction, and longer‐term resilience, this paper reviews publicly available climate information in the form of storm forecasts and storm tracks, disaster data, and a qualitative dataset on perspectives on disaster risk communication within the region. It highlights that, on average, countries often have less than 24 hours to prepare for storm impact, and contextualises this within the finding that representatives of meteorological and disaster agencies in the region indicate that there are limitations to capacity for and communication of storm risk on weekends as compared to weekdays.

These key results signal the need for increased national and subnational efforts geared towards timely and appropriate storm risk communication in relation to disaster preparedness. Alongside the importance of understanding where people may be at risk, who are particularly vulnerable, and how to mobilise action, consideration of the ‘when’ of disaster communication may also be essential. Knowledge of storm awareness times could prove valuable to meteorological offices in terms of how storms are monitored and the process via which forecasts are reviewed, authorised, and issued for public guidance. Clearer and more open lines of communication between the meteorological services and disaster agencies could help to maximise such short awareness times, and perhaps new ways of introducing ‘systems of interest’ to the public could be discussed, such that awareness is increased ahead of official recommendations and advisories. Here, while we are aware of the provision of ongoing training by the NHC to local meteorological services, further dialogue between both parties should be encouraged to assess better impending threats.

By way of example of increased national efforts that could further benefit from knowledge of storm awareness times, we review how the Cayman Islands is utilising climate information in a more timely and strategic fashion, as shared at the American Meteorological Society's 102^nd^ Annual Meeting on 23–27 January 2022. In learning from its response during the 2020 hurricane season, the 2021 mandate of the Cayman Islands National Weather Service was to ‘[p]rovide early warning and impacts of weather systems to improve person's response time so as to protect lives and property’ (Laing et al., 2022). To ensure ample time for response, the National Weather Service began to issue notifications on systems well in advance of arrival time, within various modes of communicating such impact‐based forecast notifications, such as video presentations, documents drafted for media companies to broadcast, and social media posts, including via its mobile app. Briefings were also held with Hazard Management Cayman Islands and the National Emergency Operations Committee, with meetings and notifications happening more frequently at the onset of storm events. And regular briefings were also held remotely to provide stakeholders with timely and detailed information to prepare for the events, using Zoom. Although the Cayman Islands was not one of the countries within this dataset, other countries might benefit from the lessons it has learnt and combine these procedures with respective country storm awareness times to strategise better on the issuance of notifications and collaboration with the various national stakeholders.

In terms of suggestions for other actors within the preparedness process, since local authorities play a role in confirming advisories ahead of official guidance, perhaps enhanced contingency plans for absences could be devised to minimise delays, such as on weekends or holidays. Disaster agencies can benefit from this knowledge of short awareness times through more in‐depth research on local circumstances to understand, for instance, how current preparedness plans could be activated and actualised within the average awareness period for the respective country. Doing so could proactively highlight gaps and challenges, some of which may be easy fixes, but perhaps there may be problems that require a depth of scope. There may also be factors and measures that need to be placed ahead of activating certain preparedness plans. For example, planning for processes in the short term, such as drain cleaning and tree cutting, and decisions on suppliers of certain equipment and the storage of food, water, and other resources, inter alia, would be important prior to the hurricane season, while awareness of by‐the‐hour steps by various agencies ahead of impact could improve effective preparedness and response.

In addition, specific preparedness strategies should be devised for sudden impacts in the form of zero‐lead events, with more research done to understand better the causes, circumstances, and outcomes of such events. Similarly, considering that about 21 per cent of the total observations were false alarms, that is, storm advisories issued but no approach/landfall (see Table 1), creative ways to address storm preparedness and awareness are critical to maintain public motivation to adhere to guidelines. Perhaps members of the public would benefit from *appropriate public awareness campaigns* to educate them about disaster preparedness strategies in the context of short awareness times to impel their decision‐making processes. Here, a focus on *how best to communicate disaster and climate information* is vital, as it is essential that such information is communicated in a non‐technical manner. While there will inevitably be unexpected stresses in relation to these proposals, planning based on insights from this research could assist in *preparing for preparedness* and increasing the transparency of risk reduction strategies by the various agencies involved.

The findings of this research could also be a spur to improve awareness, availability, and utility of broader climate information sources and datasets within the region (Dookie, Enenkel, and Spence, 2019). Since the timely communication of information can result in a more proactive rather than reactive approach, enriched climate information could assist the design and use of enhanced information‐related protocols, including early warning systems. Certain short‐term climate information (see Figure 1), such as a storm forecast or information on heavy rainfall or wind speed, for instance, can be disseminated to all relevant agencies as soon as a country's meteorological service starts tracking a system. Other agencies can then activate their early warning or similar system so that the information travels quickly to a wide cross‐section of agencies for resource mobilisation.

An example of an effective early warning system can be found in Jamaica. The system was set up by the Water Resources Authority in April 2019 and is meant to inform the National Works Agency and other entities of possible flooding in the Bog Walk Gorge. Since flooding mostly results from neighbouring overflow, not rainfall in the gorge, persons have become trapped in this area in the past, even resulting in loss of life. Under the early warning system, a trigger of river water levels (stage height) of six feet sends a signal indicating that inundation of the roadway within the Bog Walk Gorge at Raby's Corner has taken place. (Signals continue to be sent at every 0.5‐foot interval as the level rises or falls; if the river flood level fails to exceed 3.9 feet, then the warnings will cease.) This trigger prompts the closure of gates to prevent persons from driving through the area (resulting in road rerouting during the closure), and alerts the fire brigade, police, parish council, Meteorological Service of Jamaica, and Office of Disaster Preparedness and Emergency Management of the need for relevant action. The system was first tested in September 2019 when a surface to upper‐level trough maintained by the presence of two tropical cyclones, Jerry and Karen, produced significant rainfall that resulted in landslides and flooding. Activation of the system allowed for the closure of the entry and exit gates as well as inspection by police and fire personnel to ensure that no one was trapped in the gorge. The system was activated again in October and November 2020 when systems Zeta and Eta impacted the island, resulting in landslides in the gorge, and in August 2021 during the passage of Tropical Cyclone Grace when the Rio Cobre burst its banks.

A central system or a Common Alerting Protocol (CAP) that disseminates information alongside the Met Office could be of value to some countries, perhaps particularly smaller islands with limited capacity. Introduced by the WMO and now widely used in other countries, CAP is an international standard format for emergency alerts and public warnings designed for all hazards and all media types.^12^ While it has been set up in some islands, including Jamaica, it is not yet fully utilised, and more training is needed to allow for full‐scale implementation. Although it was introduced within a meteorological context, it is possible for different agencies to communicate tailored information in the standard format. For instance, in the event of an impending storm threat, a Met Office could send out a CAP message about the timing of the approaching system and details on it, while the relevant disaster agency could send out a different but related CAP message to affected communities regarding possible access to the nearest shelters, and the transport agency could send out a CAP message about damaged or threatened roads. These messages can be sent using various channels, including SMS (short message service) and digital and social media. As the regional authority for disaster preparedness and emergency management in the Caribbean, the CDEMA could assist countries further in this regard, geared towards more widespread uptake of CAP. At the same time, the role of the community in such messaging and communication is critical to ensure that the key messages are received and understood by the widest swath of people.

The potential design and application of a range of trigger‐based actions through forecast‐based financing strategies and wider forecast‐based early action and anticipatory actions are the subject of mounting literature (Wilkinson et al., 2021a). We reiterate here that it is apparent that frameworks that link forecasts with actions, including financing and targeting assistance to vulnerable groups, can be more efficient at getting resources to where they are needed in a timelier and more directed way. However, ‘[e]arly warning and early action also require high‐quality, accurate data to be collected and analysed for risk‐informed impact forecasts and targeted‐response action plans’ (Skorzus and Chan, 2021). Since we note that only a sample of Caribbean stakeholders felt that storm early warning systems and risk assessments were effective (Lumbroso, Brown, and Ranger, 2016), and that verification of available and relevant climate information for the region is incomplete (Dookie, Enenkel, and Spence, 2019), we suggest that further attention and research is required in this regard to ensure the requisite inputs to trigger‐based actions.

While the suggestions above are motivated by the findings of the quantitative dataset, based on the responses from the qualitative survey, it is acknowledged that although climate information is seen as relevant, there are problems with using it in the region. In particular, there is a need to improve institutional support and the availability of financial resources to utilise this information better. Furthermore, as a collection of small islands, the Caribbean is all too familiar with the challenges of limited capacity. The survey respondents also underscored that to assist capacity, enhanced technical skills were needed as well as sufficient human personnel. At the institutional level, there were concerns about having to fit new strategies and goals into existing policy and strategy frameworks. Although many of these contexts are unique to the Caribbean as vulnerable Small Island Developing States, ongoing research identifies and responds to the barriers faced by governments in accessing and operationalising technologies that support effective and cost‐efficient disaster risk management. A recent paper by the Tony Blair Institute for Global Change (Skorzus and Chan, 2021) identifies three challenges faced by governments in ‘leveraging technology and data to transform disaster management: (i) issues of governance; (ii) failure to sufficiently utilise regional and international networks; and (iii) relative lack of technical expertise and appropriate tech infrastructure’. The recommendations to respond to them are to:

- ‘Build comprehensive institutional frameworks that maximise the potential impact of technology on disaster management, set clear data‐led triggers for action and assign distinct roles and responsibilities across government;
- Build a scalable tech‐infrastructure base: a comprehensive multi‐hazard early warning system informed by multiple data sources, integrating contributions from international and regional organisations and the private sector; [and]
- Build the technical skills across government to interpret and analyse data and turn findings into action’ (Skorzus and Chan, 2021).

Although these recommendations seem necessarily apparent, if and how national governments will react to and actualise them requires additional insights, especially since each country's situation will be different depending on local circumstances and priorities. As such, there is a general need to create a broader awareness of climate information among a wider range of stakeholders, designed to instil a proactive approach to disasters, focusing more on preparedness rather than on response. Perhaps a precursor to this would be more dialogue with political decision‐makers and the private sector within each country on the role of climate information, as well as the gaps and challenges that prevent the efficient flow of information for preparedness in times of disaster.

## Conclusion

This research set out to analyse observational evidence to understand storm awareness times within 14 selected countries of the Caribbean, in the period between 1995 and 2015, as well as the perspectives of meteorological and disaster agencies on how disaster risk information is communicated within the region and some of the challenges to information awareness and dissemination. The paper highlights relatively short storm awareness times of less than 24 hours on average in the region, which may test preparedness strategies within countries. Furthermore, since there is a likely ‘weekend effect’, that is, agency capacity to assimilate and disseminate risk information may be less on a weekend as compared to a weekday, such short awareness times indicate a challenge to effective preparedness. Such findings are new in that the datasets of storm forecasts and tracks have not been collated in this way before, especially for the Caribbean, and these insights for preparedness come at a time of ongoing debates on resilience and ‘building back better’ within the region.

Through a discussion of the data and by examining some of the implications of the results, the paper offers a few suggestions regarding how such storm awareness times could be utilised best by meteorological services and disaster agencies to help streamline preparedness strategies. It also underlines the need to improve the scope, availability, and verification of climate information in general within the region, given its relevance for trigger‐based actions, including forecast‐based financing and anticipatory action. The paper notes, too, how governments worldwide are challenged in utilising data for disaster management more effectively, citing recommendations of the Tony Blair Institute for Global Change (Skorzus and Chan, 2021). For vulnerable Small Island Developing States, such as those in the Caribbean, the pressure of prioritising preparedness in the wider context of sustainable development and the limitations to capacity and availability of financial, human, and technical resources is not forgotten. However, given a changing climate and the probability of more frequent and intense storms, the time to encourage improved awareness and the utilisation of climate information, especially for disaster preparedness, is now.

## Acknowledgements

Denyse S. Dookie acknowledges the support of the Grantham Research Institute on Climate Change and the Environment at the London School of Economics and Political Science, and the Economic and Social Research Council's Centre for Climate Change Economics and Policy (ref. ES/R009708/1). Many thanks to Dr Arlene Laing of the Caribbean Meteorological Organization for her insights, Dr Daniel Osgood and Professor Declan Conway for their reviews of earlier drafts of this paper, and the three anonymous peer reviewers for their suggestions and comments. We also value the time and insights of the responding participants at the 2016 CariCOF, and the assistance provided by Ashley Curtis and Cédric van Meerbeck with the survey.

## Data availability statement

The data that support the findings of this study are available from the corresponding author upon reasonable request.

## Endnotes

1 For more information, see https://www.emdat.be (data as of 3 November 2021; website last accessed on 4 May 2022).

2 For more information, see https://cdema.org/rtc/ (last accessed on 4 May 2022).

3 For more information, see https://rcc.cimh.edu.bb/caricof/ (last accessed on 4 May 2022).

4 For more information, see https://www.nhc.noaa.gov/watchwarn_changes.shtml (last accessed on 4 May 2022).

5 For more information, see https://www.nhc.noaa.gov/ (last accessed on 4 May 2022).

6 HURDAT2 refers to a NHC Atlantic Hurricane database: HURricane DATa 2^nd^ generation. For more information, see https://www.nhc.noaa.gov/data/hurdat/hurdat2-format-atl-1851-2021.pdf (last accessed on 26 May 2022).

7 IBTrACS refers to the International Best Track Archive for Climate Stewardship project, which is the most complete global collection of tropical cyclones available. It merges recent and historical tropical cyclone data from multiple agencies to create a unified, publicly available, best‐track dataset that improves inter‐agency comparisons. For more information, see https://www.ncei.noaa.gov/products/international-best-track-archive (last accessed on 26 May 2022).

8 For more information, see https://coast.noaa.gov/hurricanes/ (last accessed on 4 May 2022).

9 For more information, see https://public.emdat.be/ (last accessed on 4 May 2022).

10 For more information, see https://www.nhc.noaa.gov/aboutintro.shtml (last accessed on 9 May 2022).

11 While the analysis of the data specifically focused on weekends, we also asked about holidays in the survey question, as a follow‐up aspect to the research. For the purpose of this paper, however, the weekend component is more essential to understanding.

12 For more information, see https://www.fema.gov/emergency-managers/practitioners/integrated-public-alert-warning-system/technology-developers/common-alerting-protocol (last accessed on 26 May 2022).

## References

[disa12540-bib-0001] Belles, J. (2017) ‘5 changes coming to hurricane season forecasts’. The Weather Channel website. 19 March. https://weather.com/storms/hurricane/news/changes-coming-this-hurricane-season-2017 (last accessed on 9 May 2022).

[disa12540-bib-0002] Bello, O.D. (2017) ‘Disasters, economic growth and fiscal response in the countries of Latin America and the Caribbean, 1972–2010’. CEPAL Review. 121(April). pp. 7–29.

[disa12540-bib-0003] Bello, O.D. , M. Khamis , C. Osorio , and L. Peralta (2017) Mainstreaming Disaster Risk Management Strategies in Development Instruments: Policy Briefs for Selected Member Countries of the Caribbean Development and Cooperation Committee. LC/TS.2017/80. United Nations, Santiago.

[disa12540-bib-0004] Burn, M.J. and S.E. Palmer (2015) ‘Atlantic hurricane activity during the last millennium’. Scientific Reports. 5. Article number: 12838. 10.1038/srep12838. PMC4525293 26243340

[disa12540-bib-0005] Camargo, S.J. , F. Vitart , C.-Y. Lee , and M.K. Tippett (2021) ‘Skill, predictability, and cluster analysis of Atlantic tropical storms and hurricanes in the ECMWF monthly forecasts’. Monthly Weather Review. 149. pp. 3781–3802.

[disa12540-bib-0006] Campbell, J.D. , M.A. Taylor , T.S. Stephenson , R.A. Watson , and F.S. Whyte (2011) ‘Future climate of the Caribbean from a regional climate model’. International Journal of Climatology. 31(12). pp. 1866–1878.

[disa12540-bib-0007] Charvériat, C. (2000) Natural Disasters in Latin America and the Caribbean: An Overview of Risk. IDB Working Paper No. 364. 10.2139/ssrn.1817233.

[disa12540-bib-0008] Chmutina, K. and L. Bosher (2015) ‘Disaster risk reduction or disaster risk production: the role of building regulations in mainstreaming DRR’. International Journal of Disaster Risk Reduction. 13 (September). pp. 10–19.

[disa12540-bib-0009] Collymore, J. (2011) ‘Disaster management in the Caribbean: perspectives on institutional capacity reform and development’. Environmental Hazards. 10(1). pp. 6–22.

[disa12540-bib-0010] Dinku, T. , K. Asefa , K. Hilemariam , D. Grimes , and S. Connor (2011) ‘Improving availability, access and use of climate information’. Bulletin. 60(2). https://public.wmo.int/en/bulletin/improving-availability-access-and-use-climate-information (last accessed on 9 May 2022).

[disa12540-bib-0011] Dookie, D.S. , M. Enenkel , and J. Spence (2019) ‘From science to science-based: using state-of-the-art climate information to strengthen DRR in small island states’. In W.H. Khonje and T. Mitchell (eds.) Strengthening Disaster Resilience in Small States. Commonwealth Secretariat, London, pp. 13–41.

[disa12540-bib-0012] Dookie, D.S. and D. Osgood (2020) ‘Rainy days on Mondays: storm proxies, human actions and disaster outcomes in the Caribbean’. SSRN Electronic Journal. 1 December. 10.2139/ssrn.3759941.

[disa12540-bib-0013] Dookie, D.S. and D. Osgood (2021) ‘Widening the scope of disaster preparedness in Caribbean small island developing states (SIDS): building resilience through improving climate information’. In S. Moncada (ed.) Small Island Developing States: Vulnerability and Resilience under Climate Change. Springer Nature, Cham. pp. 81–111.

[disa12540-bib-0014] Emanuel, K. (2017) ‘Will global warming make hurricane forecasting more difficult?’. Bulletin of the American Meteorological Society. 98(3). pp. 495–501.

[disa12540-bib-0015] Heger, M. , A. Julca , and O. Paddison (2008) Analysing the Impact of Natural Hazards in Small Economies: The Caribbean Case. Research Paper 2008/025. March. United Nations University–World Institute for Development Economics Research, Helsinki.

[disa12540-bib-0016] Hsiang, S.M. (2010) ‘Temperatures and cyclones strongly associated with economic production in the Caribbean and Central America’. Proceedings of the National Academy of Sciences. 107(35). pp. 15367–15372. 10.1073/pnas.1009510107 PMC2932627 20713696

[disa12540-bib-0017] Kirton, M. (2013) Caribbean Regional Disaster Response and Management Mechanisms: Prospects and Challenges. The Brookings Institution, Washington, DC.

[disa12540-bib-0018] Knutson, T.R. et al. (2010) ‘Tropical cyclones and climate change’. Nature Geoscience. 3 (March). pp. 157–163.

[disa12540-bib-0019] Laing, A. et al. (2022) ‘Responding to multiple hazards in the Caribbean: volcanic eruptions, heavy rainfall, hurricanes and an earthquake’. Paper for the American Meteorological Society's 102^nd^ Annual Meeting on 23–27 January 2022. Abstract available at https://ams.confex.com/ams/102ANNUAL/meetingapp.cgi/Paper/399989 (last accessed on 31 May 2022).

[disa12540-bib-0020] Landsea, C. (1993) ‘A climatology of intense (or major) Atlantic hurricanes’. Monthly Weather Review. 121(6). pp. 1703–1713.

[disa12540-bib-0021] Landsea, C. and J.P. Cangialosi (2018) ‘Have we reached the limits of predictability for tropical cyclone track forecasting?’. Bulletin of the American Meteorological Society. 99(11). pp. 2237–2243.

[disa12540-bib-0022] López-Marrero, T. and B. Wisner (2012) ‘Not in the same boat: disasters and differential vulnerability in the Caribbean’. Caribbean Studies. 40(2). pp. 129–168.

[disa12540-bib-0023] Lumbroso, D. , E. Brown , and N. Ranger (2016) ‘Stakeholders’ perceptions of the overall effectiveness of early warning systems and risk assessments for weather-related hazards in Africa, the Caribbean and South Asia'. Natural Hazards. 84(3). pp. 2121–2144.

[disa12540-bib-0024] Mahon, R. et al. (2019) ‘Fit for purpose? Transforming national meteorological and hydrological services into national climate service centers’. Climate Services. 13 (January). pp. 14–23.

[disa12540-bib-0025] Mendelsohn, R. , K. Emanuel , S. Chonabayashi , and L. Bakkensen (2012) ‘The impact of climate change on global tropical cyclone damage’. Nature Climate Change. 2 (March). pp. 205–209.

[disa12540-bib-0026] Mohan, P. and E. Strobl (2021) ‘The impact of tropical storms on the accumulation and composition of government debt’. International Tax and Public Finance. 28 (June). pp. 483–496.

[disa12540-bib-0027] Murray, M. and P.K. Watson (2019) ‘Adoption of natural disaster preparedness and risk reduction measures by business organisations in small island developing states – a Caribbean case study’. International Journal of Disaster Risk Reduction. 39 (October). Article number: 101115. 10.1016/J.IJDRR.2019.101115.

[disa12540-bib-0028] Pelling, M. (2010) Review and Systematization of Disaster Preparedness Experiences in Urban Areas in the Caribbean Region. https://www.alnap.org/help-library/review-and-systematization-of-disaster-preparedness-experiences-in-urban-areas-in-the (last accessed on 9 May 2022).

[disa12540-bib-0029] Pielke, Jr., R.A. , J. Rubiera , C. Landsea , M.L. Fernández , and R. Klein (2003) ‘Hurricane vulnerability in Latin America and the Caribbean: normalized damage and loss potentials’. Natural Hazards Review. 4(3). pp. 101–114.

[disa12540-bib-0030] Pulwarty, R.S. , L.A. Nurse , and U.O. Trotz (2010) ‘Caribbean islands in a changing climate’. Environment: Science and Policy for Sustainable Development. 52(6). pp. 16–27.

[disa12540-bib-0031] Rasmussen, T.N. (2004) Macroeconomic Implications of Natural Disasters in the Caribbean. IMF Working Papers WP/04/224. 10.5089/9781451875355.001.

[disa12540-bib-0032] Regnier, E.D. (2020) ‘What is six hours worth? The impact of lead time on tropical-storm preparation decisions’. Decision Analysis. 17(1). pp. 9–23.

[disa12540-bib-0033] Robinson, C.J. and P. Bangwayo‐Skeete (2016) ‘The financial impact of natural disasters: assessing the effect of hurricanes and tropical storms on stock markets in the Caribbean’. SSRN Electronic Journal. 16 March. 10.2139/SSRN.2845429.

[disa12540-bib-0034] Singh, C. et al. (2018) ‘The utility of weather and climate information for adaptation decision-making: current uses and future prospects in Africa and India’. Climate and Development. 10(5). pp. 389–405.

[disa12540-bib-0035] Skorzus, R. and K. Chan (2021) A Roadmap for Managing Disasters: How Climate-vulnerable Countries can Access Tech. 16 December. Tony Blair Institute for Global Change, London.

[disa12540-bib-0036] Spencer, N. and M.-A. Urquhart (2018) ‘Hurricane strikes and migration: evidence from storms in Central America and the Caribbean’. Weather, Climate, and Society. 10 (July). pp. 569–577.

[disa12540-bib-0037] Strobl, E. (2012) ‘The economic growth impact of natural disasters in developing countries: evidence from hurricane strikes in the Central American and Caribbean regions’. Journal of Development Economics. 97(1). pp. 130–141.

[disa12540-bib-0038] UNDRR (United Nations Office for Disaster Risk Reduction) (2022a) ‘Terminology: build back better’. Website. https://www.undrr.org/terminology/build-back-better (last accessed on 9 May 2022).

[disa12540-bib-0039] UNDRR (2022b) ‘Terminology: preparedness’. Website. https://www.undrr.org/terminology/preparedness (last accessed on 9 May 2022).

[disa12540-bib-0040] UN OCHA (United Nations Office for the Coordination of Humanitarian Affairs) (2020) Natural Disasters in Latin America and the Caribbean: 2000–2019. UN OCHA, New York, NY.

[disa12540-bib-0041] Upperman, C.R. , G. Marcelle , and C. Excell (2019) ‘Strengthening disaster preparedness in the Caribbean’. World Resources Institute website. 21 October. https://www.wri.org/insights/strengthening-disaster-preparedness-caribbean (last accessed on 9 May 2022).

[disa12540-bib-0042] Vermeiren, J.C. and C.C. Watson, Jr . (1994) ‘New technology for improved storm risk assessment in the Caribbean’. Disaster Management. 6(4). pp. 1–6.

[disa12540-bib-0043] Walker, L.R. , D.J. Lodge , N.V.L. Brokaw , and R.B. Waide (1991) ‘An introduction to hurricanes in the Caribbean’. Biotropica. 23(4a). pp. 313–316.

[disa12540-bib-0044] Wilkinson, E. , J. Twigg , and R. Few (2018) Building Back Better: A Resilient Caribbean after the 2017 Hurricanes. Briefing note. January. Overseas Development Institute, London.

[disa12540-bib-0045] Wilkinson, E. et al. (2021a) Preparing for Extreme Weather in the Eastern Caribbean: What Role for Forecast-based Early Action? Working Paper 603. March. Overseas Development Institute, London.

[disa12540-bib-0046] Wilkinson, E. et al. (2021b) Enhanced Preparedness for Extreme Weather Across the Caribbean: A Joint Work Plan. March. Overseas Development Institute, London.

[disa12540-bib-0047] WMO (World Meteorological Organization) (2011) WMO Strategic Plan: 2012–2015. WMO-No. 1069. WMO, Geneva.

